# Microcrack healing in non-ferrous metal tubes through eddy current pulse treatment

**DOI:** 10.1038/s41598-018-24354-7

**Published:** 2018-04-16

**Authors:** Wenchen Xu, Chuan Yang, Haiping Yu, Xueze Jin, Bin Guo, Debin Shan

**Affiliations:** School of Materials Science and Engineering & National Key Laboratory for Precision Hot Processing of Metals, Harbin Institute of Technology, Harbin, 150001 China

## Abstract

This study proposed a novel method to heal microcrack within Mg alloy tubes using high density eddy current pulse treatment (ECPT). Through electromagnetic induction inside a copper coil connected with a high density pulse power source supply, the high density (greater than 5 × 10^9^ A/m^2^) and short duration eddy current was generated in tube specimens of Mg alloy. The results show that the microcracks in tube specimens was healed evidently and the mechanical properties of the tubes subjected to ECPT were improved simultaneously. The crack healing during ECPT was ascribed to not only the thermal stress around the microcrack tips and the softening or melting of metals in the vicinity of microcrack tips, but also the squeezing action acted by the Lorentz force. In the inward-discharging scheme, both the compressive radial stress and tangential stress induced by the Lorentz force contributed to more sufficient crack healing and thus better mechanical properties of tube specimens after the ECPT experiment, compared to the outward-discharging scheme. The ECPT can heal microcracks automatically without directly contacting tubular specimens and is not limited by the length of tubular workpieces, exhibiting great potential for crack healing in non-ferrous alloy tubes.

## Introduction

Degradation, damage and failure are natural consequence of materials^1^. The degradation usually occurs in organic and inorganic materials, while the damage appears in metallic materials besides organic and inorganic materials. In terms of structural materials, the degradation and damage are prone to microcracks and then cause a failure. As is known, the failure of materials often leads to severe accidents and huge losses in engineering applications. If the cracks can be healed or repaired in intermediate or even micro scale, the reliability and lifetime of structural materials will be enhanced pronouncedly. Hence, the study on crack healing is garnering extensive interest from materials scientists. To date, there have been great advances in the crack healing of biological materials, polymers and ceramics, wherein the crack healing methods include vascular^1^ or hollow fiber^2^ method, microcapsule method^3^, SMA (shape memory alloy) method^4^, plasma coating method^5^, etc. But as far as the metals are concerned, it is much more difficult to heal cracks than for other materials owing to high bond strength, small volumes and low diffusion rates of metallic atoms^6^, so there has been very limited progress in damage healing of metallic materials until now. Olson G.B^7^. showed that shape memory alloy (SMA) wires could repair the cracks in off-eutectic metal matrices. Lumley R.N^8^. demonstrated that dynamic precipitation could reduce the cracks and voids in the supersaturated solid solution of Al alloy. However, SMA wires and dynamic precipitation techniques could not be used for pre-existing structures because the SMA wires and precipitation elements must be added into the materials in advance^9^. Yu H.L. *et al*.^10^ reported that the internal cracks in low-carbon steel could be healed by hot plastic deformation. Wei D. *et al*.^11^ indicated that crack healing in crack tips could be achieved after heat treatment at high temperature. But hot plastic deformation and heat treatment techniques may significantly change the microstructure and performance of materials.

In recent years, the high density current pulse has been employed to heal the cracks in stainless steel^12,13^, 1045 steel^14,15^ and titanium alloy^16^. The healing effect can be ascribed to the melting and thermal compressive stress around crack tips, which is caused by the detouring of pulse current around the cracks. The temperature increase around the crack could also enhance the fracture resistance of specimen, which is known as the warm prestressing effect^17,18^ proposed as early as 1980s. The high density current pulse treatment can automatically detect the locations of cracks and quickly heal the crack without changing the microstructure of materials obviously^19^, which shows great application potential in metallic materials. Nevertheless, current researches on crack healing by high density current are mostly concentrated in simple sheet samples, while the crack healing in more complex-shaped components, such as tube specimens, has been seldom reported until now. As one kind of important structural components, thin-walled tubes are widely used in various fields, such as chemistry, power, oil and gas, aviation and aerospace industries. The damage and microcrack can decrease burst pressure, longitudinal and lateral load-capacity of tube components and even cause catastrophic failure in advance, so the study on the healing of cracks inside metal tubes is of great significance in engineering application.

Because of their complex geometries, it is not convenient to directly apply electropulsing treatment to the tube components since the electrodes holding tube ends may bring about electric sparking in the contact regions between the tube ends and electrodes. The surface of electrodes or tube specimens is not so smooth, so the air gaps exist between the surface of electrodes and tube specimens. And the air gap may be broken down by high voltage, thus inducing electric sparks. Besides, this method is inappropriate for healing cracks inside super long tubes in practical application. Therefore, we firstly proposed the eddy current treatment to heal the cracks in metallic materials recently^20^. During the treatment process, the eddy current in the form of electric current loops was induced within conductors through the induction coil, and the experimental results presented evident crack healing effect after eddy current treatment^20^. Moreover, this method does not apply high density current to the tube specimen directly, exhibiting high efficiency and safe working condition. However, amounts of high-strength and light-weight nonferrous alloys widely used in automobile and aviation industries, such as magnesium alloys, aluminum alloys and titanium alloys, are paramagnetic or diamagnetic materials, so the eddy current density induced within those alloys attenuate severely compared to ferromagnetic carbon steel under the same condition of eddy current treatment. To address this problem, the high density (greater than 5 × 10^9^ A/m^2^) and short duration eddy current is generated through the copper coil connected with a high density pulse power source supply and applied to the tube specimens of magnesium alloy containing high content of rare earth (RE) elements in this study. After the eddy current pulse treatment (ECPT), the microcrack evolution has been observed and the mechanical properties of tube specimens have been tested. The results show remarkable effect of crack healing and mechanical property strengthening of the RE-containing magnesium alloy tubes. Through the finite element analysis (FEA), the crack healing mechanisms and the difference of various ECPT schemes have been revealed. This study firstly proposes an effective pathway to heal crack in tube specimens of paramagnetic and diamagnetic metals.

## Materials and Experimental Procedures

**Figure 1:**
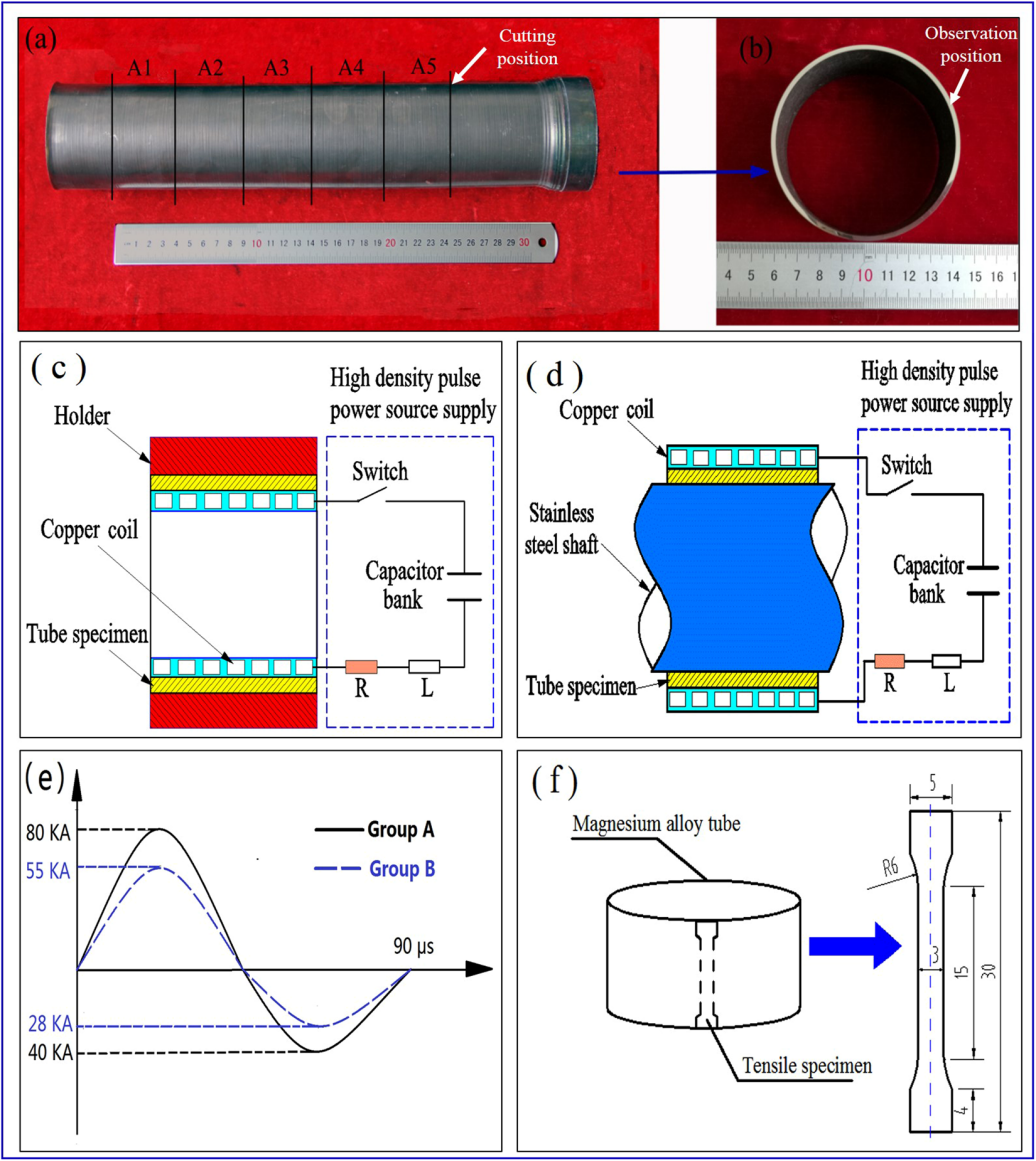
(**a**) Mg alloy as-spun tube. (**b**) specimen cut from as-spun tube. (**c**,**d**) the schematic diagrams in outward-discharging scheme (**c**) and inward-discharging scheme (**d**) under ECPT. (**e**) the exciting current waveforms in group A and B. (**f**) schematic of tensile specimen (unit: mm).

The tubes used in this study were made of as-cast magnesium alloy containing high extent of rare earth (RE) elements, which were prepared through the power spinning process^21,22^, as shown in Fig. 1a. The chemical composition of the RE magnesium alloy is listed in Table 1. The as-spun tube blank of Mg alloy was 72.3 mm in inner diameter and 8.0 mm in wall thickness. After power spinning at about 450 °C with the total thickness reduction of 77% in five passes, the as-spun tubes were thinned to 1.7 ± 0.10 mm in thickness and elongated to more than 300 mm in length. Two as-spun Mg alloy tubes were fabricated for two groups of ECPT experiments. The tube specimens for ECPT were cut from the middle area of as-spun tubes through wire electrical discharge machining (WEDM) and machined to 30 mm in length, as shown in Fig. 1b.

**Table 1:** Chemical composition of magnesium alloy (wt%).

Composition	Mg	Gd	Y	Zn	Zr	Mn	Fe
Wt%	86.46	6.84	5.06	0.62	0.96	0.04	0.02

After the morphologies of cross sections of as-spun tubes were recorded, the high density eddy current pulse treatment (ECPT) was applied to the tube specimens under ambient temperature. Both the outward-discharging (or outward) scheme and inward-discharging (or inward) scheme for ECPT were adopted, in which the tube specimens were fixed outside and inside the copper coil respectively, as shown in Fig. 1c,d. The device for ECPT had a high density pulse power source supply including a capacitor bank with the maximum discharging voltage of 15 KV, which could provide the total energy storage of about 50 kJ. The circular copper coil was connected with the power source supply, whose surface was covered by an insulation layer. The copper coils in both the ECPT schemes were about 50 mm in length, which were longer than the tube specimens in order to cover the tube specimen completely. In the outward-discharging scheme, a stainless steel holder was fixed outside the tube specimen to restrict its radial expansion (see Fig. 1c). In the inward-discharging scheme, the stainless steel shaft was fixed inside the tube specimen to restrict its radial contraction (see Fig. 1d). When the switch was on, the capacitor bank rapidly discharged and high density pulse current occurred in the copper coil, which could induce high density eddy current within the tube specimen. Table 2 shows the processing conditions of ECPT on the tube specimens in group A and B, which were cut from the two as-spun tubes, respectively. The tube specimens A1 - A4 were applied by ECPT in outward scheme with 0, 5, 10, 15 cycles at 6 KV discharging voltage of capacitor bank respectively, while the tube specimen A5 was applied by ECPT in inward scheme with 15 cycles at 6 KV discharging voltage in contrast to tube specimen A4. Similarly, the tube specimens B1 - B4 were applied by ECPT in inward scheme with 0, 5, 10 and 15 cycles at 9 KV discharging voltage of capacitor bank respectively, while the tube specimen B5 was applied by ECPT in outward scheme with 15 cycles at 9 KV discharging voltage in comparison with tube specimen B4. Here, each cycle of ECPT denoted a discharge process of capacitor bank. In the discharging experiments, the exciting current within the copper coil was measured through a current sensor, which exhibited attenuating sine waves, as shown in Fig. 1e. The tube specimens were cooled in air at the time interval of 30 seconds between every two cycles of discharge treatments in order to prevent the thermal accumulation. Before the ECPT, the cross sections of tube specimens were mechanically polished, and the morphologies of cross sections were observed by scanning electron microscopy (SEM) on Quanta 200FEG, as depicted in Fig. 1b. After the SEM observation on the initial cross section of as-spun tube specimen, the tube specimens were taken out and then treated by the ECPT cycles. In the ECPT experiment, the coil was placed inside the tube specimen in outward scheme (see in Fig. 1c) and outside the tube specimen in inward scheme (see Fig. 1d). After every five cycles of ECPT, the tube specimens were taken out for SEM observation on the cross section of tube specimen end. The specimens before and after ECPT were also observed through TEM (Transmission Electron Microscopy) analysis on Talos F200X. After the ECPT experiments, the mechanical properties of tube specimens were tested on an Instron 5569 test machine with a drawing speed of 0.8 mm/min under ambient temperature. The tensile specimens were cut from the tube specimens along the axial direction, as illustrated in Fig. 1f. Ten specimens cut from the same tube specimen were tested as a group.

**Table 2:** Treatment conditions of ECPT on the specimen in the group A and B.

Specimen	A1	A2	A3	A4	A5
Scheme	Outward	Outward	Outward	Outward	Inward
Voltage	6 KV	6 KV	6 KV	6 KV	6 KV
ECPT cycles	0	5	10	15	15
**Specimen**	**B1**	**B2**	**B3**	**B4**	**B5**
**Scheme**	**Inward**	**Inward**	**Inward**	**Inward**	**Outward**
Voltage	9 KV	9 KV	9 KV	9 KV	9 KV
ECPT cycles	0	5	10	15	15

## Results and Discussion

**Figure 2:**
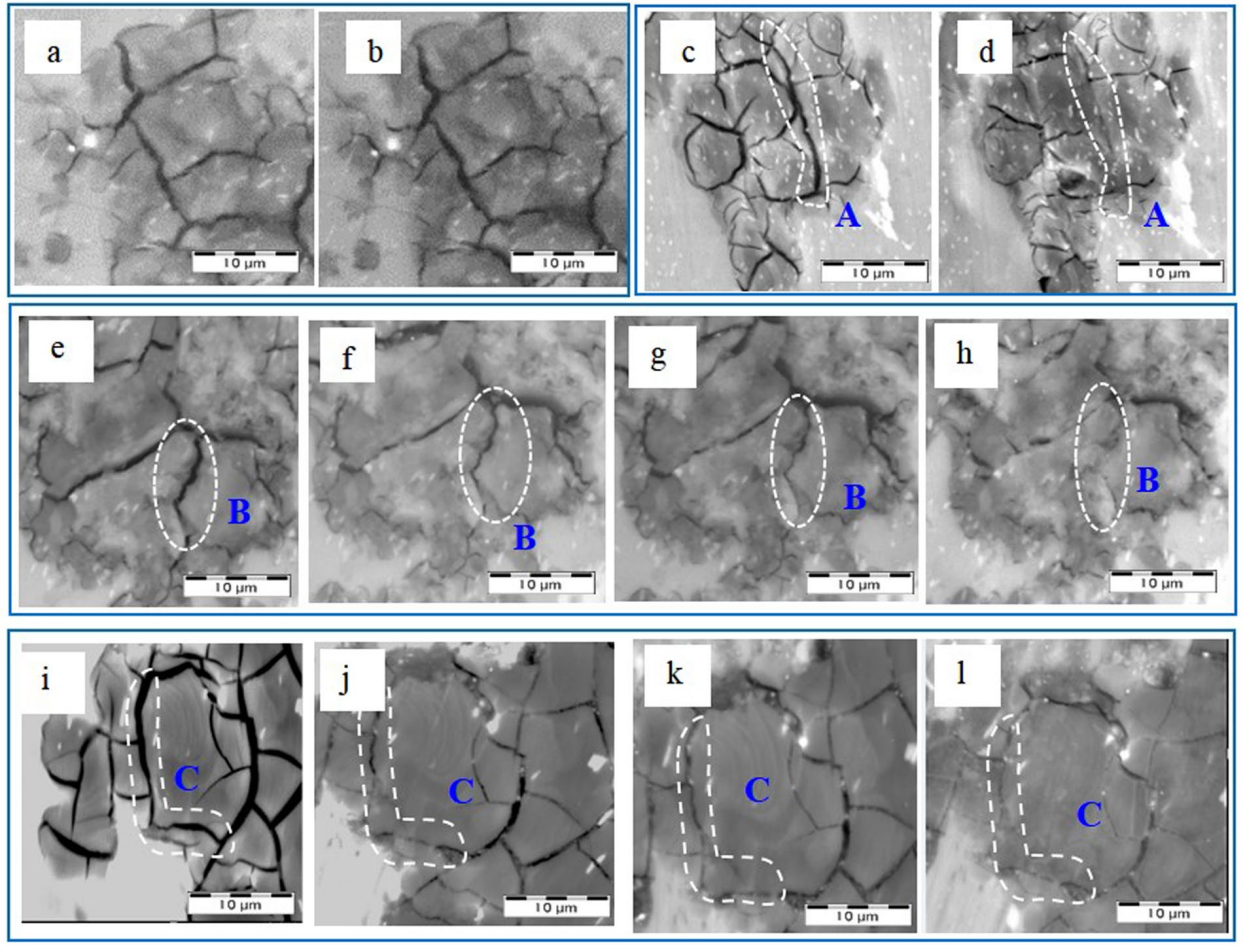
SEM images of cross sections of tube specimens (**a**,**b**) A2 before and after 5 ECPT (outward scheme); (**c**,**d**) A3 before and after 10 ECPT(outward scheme); (**e**–**h**) as-spun and after 5, 10,15 ECPT of A4 (outward scheme); (**i**–**l**) as-spun and after 5, 10,15 ECPT of A5 (inward scheme).

After spinning deformation, amounts of microcracks were observed on the cross sections of tube specimens both in group A and B since the RE-containing Mg alloy exhibited higher strength at the expense of ductility. Figure 2 shows the morphologies of microcracks on the cross sections of tube specimens before and after the ECPT. The A2-A4 were subject to the ECPT in the outward-discharging scheme. As shown in Fig. 2a,b, there was no obvious change in the morphologies of mircocracks on the cross section of specimen A2 before and after 5 cycles ECPT. However, when subjected to ECPT for 10 cycles, the mircocracks on the cross section of tube specimen A3 started to close in local areas, as illustrated in Fig. 2c,d, respectively. The microcracks were narrowed and even completely closed in region A, and the microcracks in neighboring areas also exhibited a healing tendency by degrees. The similar situation occurred when the specimen A4 was subject to ECPT for 15 cycles. In order to study the evolution of the microcracks during the ECPT, the cross section of tube specimen A4 was observed after every fifth cycle treatment, as shown in Fig. 2e–h. The SEM images taken from region B depicted the gradual narrowing and closing of microcracks on the cross section of tube specimens, indicating a remarkable effect of ECPT on microcrack healing in the magnesium alloy. Besides, the evolution of mircocracks in tube specimen A5 in the inward-discharging scheme with 6KV discharge voltage exhibited similar tendency in outward-discharging scheme. As illustrated in Fig. 2i–l, the microcracks in region C were gradually narrowed and finally closed as the ECPT cycles increased.

**Figure 3:**
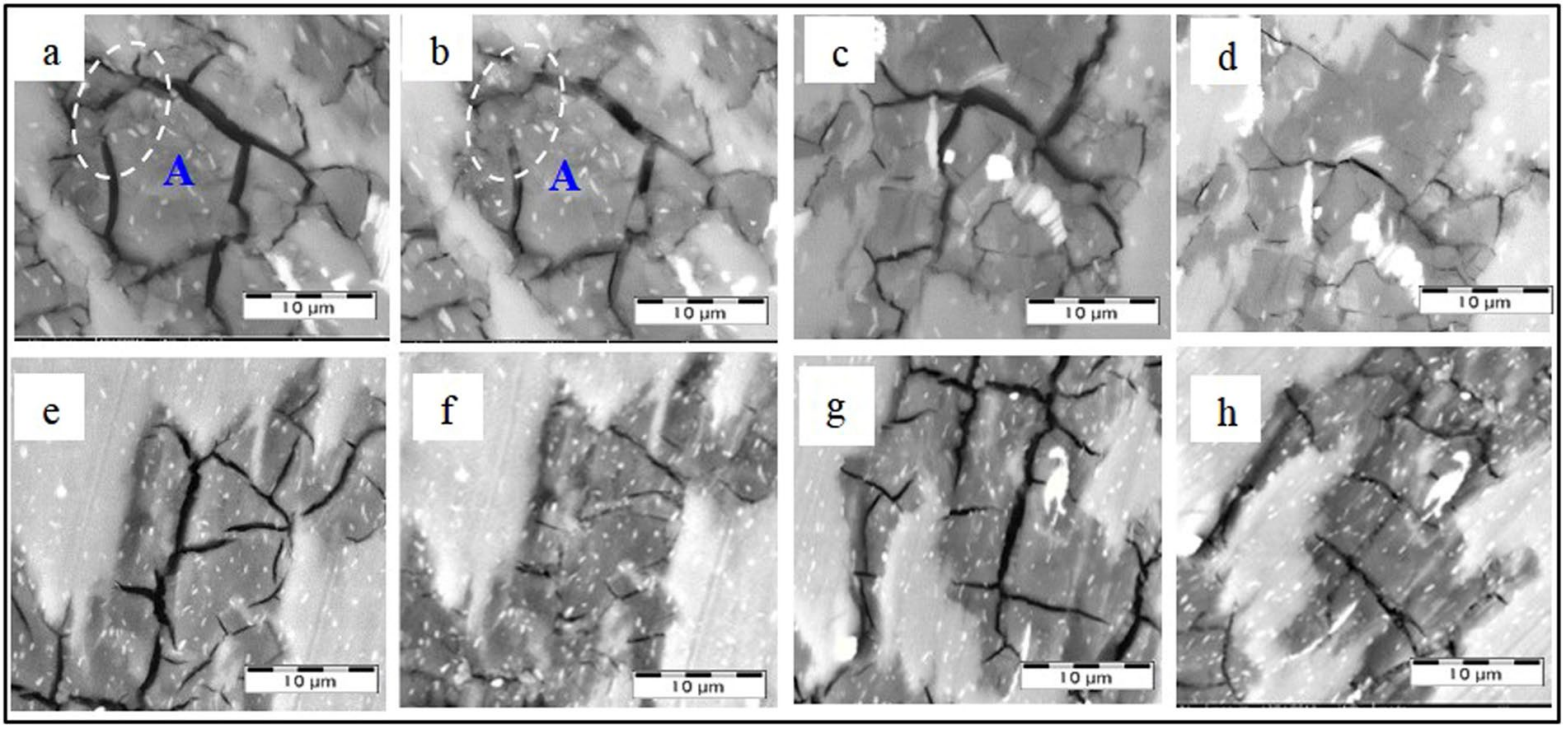
SEM images of cross section of tube specimens: (**a**,**b**) B2 before and after 5 ECPT (inward scheme); (**c**,**d**) B3 before and after 10 ECPT (inward scheme); (**e**,**f**) B4 before and after 15 ECPT (inward scheme); (**g**,**h**) B5 before and after 15 ECPT (outward scheme).

Compared to the tube specimens in group A subjected to outward-discharging treatment, the microcrack morphologies of tube specimens in group B subjected to inward discharging treatment showed similar evolution tendency. After 5 cycles of ECPT with the inward-discharging scheme at 9 KV, it can be seen from Fig. 3a,b that some local areas of microcracks in tube specimen B2, such as region A, became closed. When the ECPT increased to 10 cycles, most of the microcracks in tube specimens B3 were basically closed or pronouncedly narrowed, as shown in Fig. 3c,d. After 15 cycles of ECPT, almost all the microcracks were healed, as shown in Fig. 3e,f. For tube specimen B5 treated with 15 cycles of ECPT by the outward-discharging scheme at 9 KV, crack healing was also clearly observed, while the healing degree seems less than that for tube specimen B4 in the inward-discharging scheme.

**Figure 4:**
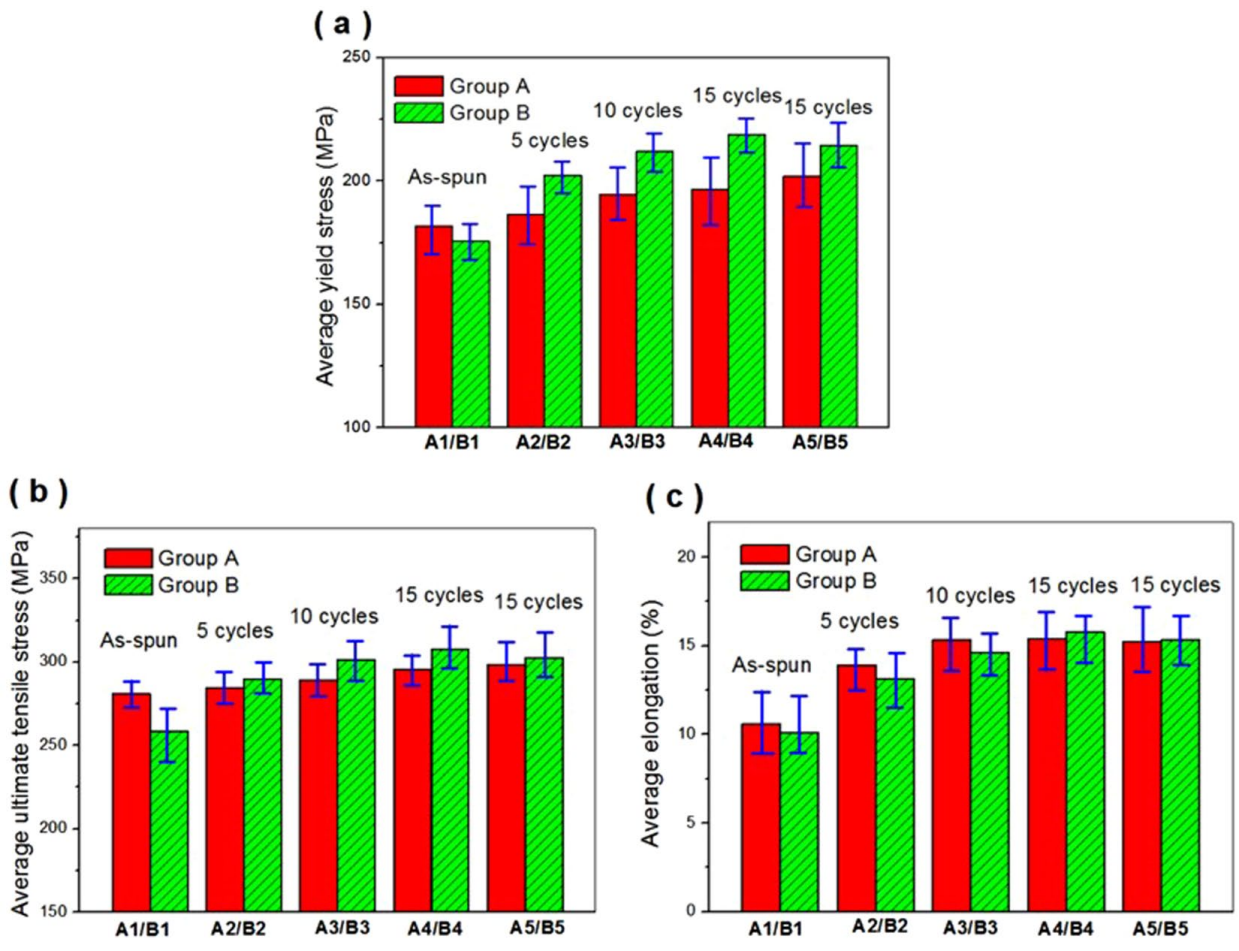
Uniaxial tensile experiment results of group A and B: (**a**) average yield stress, (**b**) average ultimate tensile stress, (**c**) average elongation.

The microcrack healing in the tube specimens may lead to the change of mechanical properties, so the mechanical properties of tube specimens were tested through uniaxial tensile experiment. Figure 4 shows the yield strength, ultimate tensile strength and elongation of tube specimens in group A and B, respectively, before and after the ECPT. The average yield strength of as-spun specimens in group A1 was 170.83 MPa, and the average yield strength of groups A2, A3 and A4 reached 182.37 MPa, 194.01 MPa and 199.13 MPa after 5, 10 and 15 cycles of ECPT respectively, increasing by 6.75%, 13.86% and 16.56% respectively (see Fig. 4a). It indicates that the average yield stresses gradually increased with the increasing cycles of ECPT, while it was not so significant when the ECPT reached more than 10 cycles. Also, the ECPT improved the ultimate tensile stress of as-spun tubes with increasing ECPT cycles, and the average values of group A2, A3 and A4 increased by 5.23%, 10.66%, 12.76%, respectively, compared to that of initial as-spun tubes A1 (280.91 MPa), as illustrated in Fig. 4b. The average elongation of group A1 was 10.37%, while the average elongation of group A2, A3, A4 reached 12.68%, 15.03% and 15.87%, enhanced by 22.26%, 44.94% and 53.04%, respectively. Clearly, the elongation was significantly improved with increasing treatment cycles. Therefore, the mechanical properties were gradually promoted with the increasing cycles of ECPT, but it became less remarkable as the ECPT surpassed 10 cycles in outward-discharging scheme. In order to compare the strengthening effect of ECPT with outward-discharging scheme at 6 KV, the specimen A5 was subjected to ECPT with 15 cycles using inward-discharging scheme under the same discharging voltage, and the average yield strength, ultimate tensile strength and elongation of specimen A5 increased to 202.12 MPa, 298.47 MPa, 16.11% respectively, which were slightly higher than those of specimen A4 (193.96 MPa, 294.24 MPa, 15.24%) subjected to ECPT with 15 cycles using outward-discharging scheme.

For the specimens in group B, the average yield strength of B2, B3 and B4 increased to 199.55 MPa, 212.41 MPa and 215.23 MPa (see Fig. 4a), respectively, which rose by 12.94%, 22.26% and 23.89%, respectively, compared to specimens B1 (173.73 MPa). The average ultimate tensile strength of initial specimen B1 was 263.02 MPa, which increased by 10.79%, 15.44% and 20.02%, respectively, after 5, 10 and 15 cycles of ECPT (see Fig. 4b). The average elongation of B2, B3 and B4 increased by 31.37%, 50.84% and 62.77%, respectively, compared to specimen B1 (10.03%). When subject to ECPT at 9 KV, the specimen B5 treated by 15 cycles using outward-discharging scheme had the average yield strength (215 MPa), ultimate tensile strength (311.33 MPa) and elongation (15.35%), respectively, which were slightly smaller than the values of the specimen B4 treated by 15 cycles using inward-discharging scheme (see Fig. 4a–c). Therefore, it can be noted that the specimens treated with inward-discharging scheme exhibited better mechanical performance than those with outward-discharging scheme.

**Figure 5:**
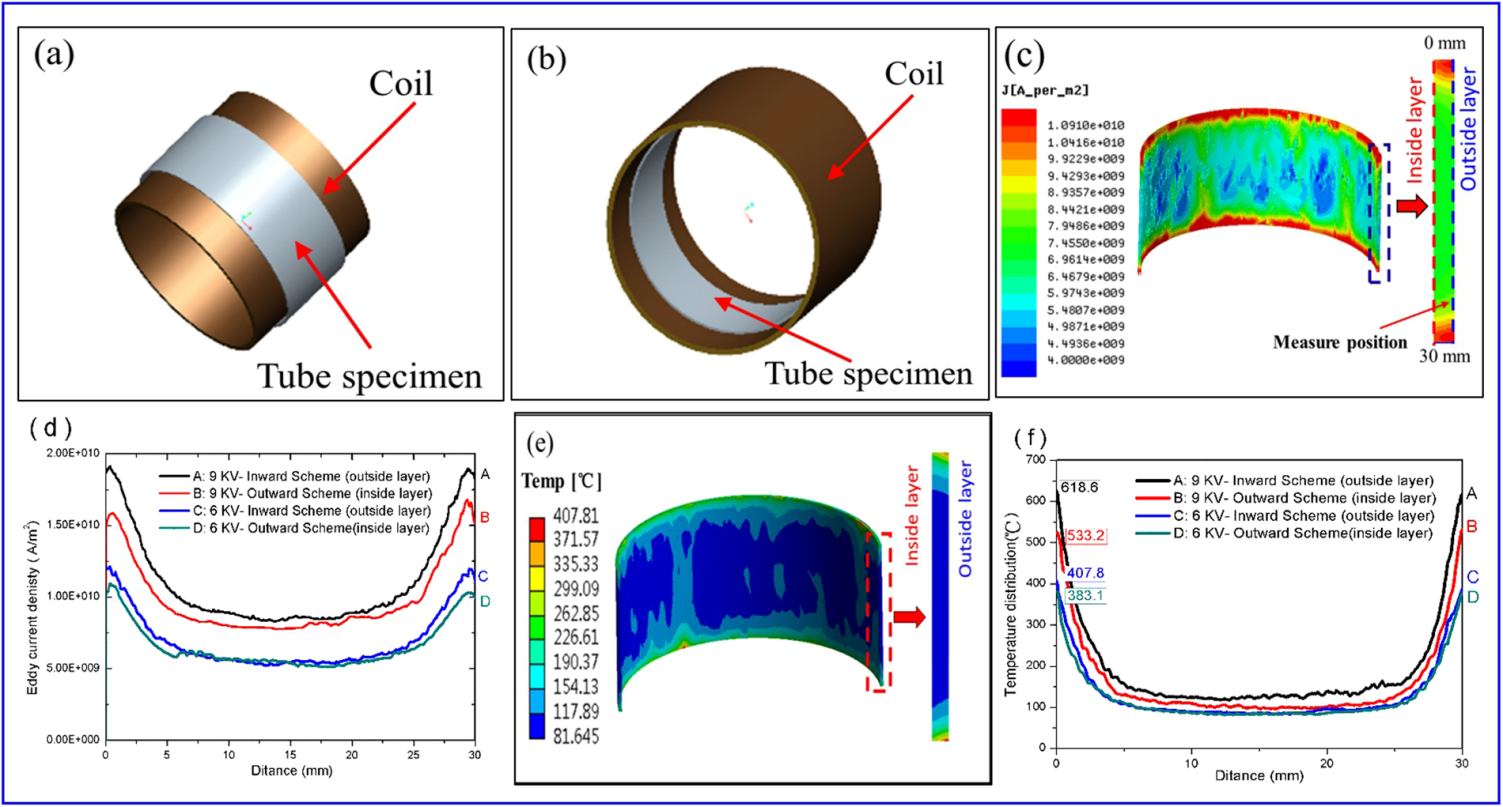
FEM results of ECPT (t = 90 μs): (**a**) model in outward scheme, (**b**) model in inward scheme; (**c**) distribution of eddy current density induced within the tube specimens in inward scheme A at 6 KV; (**d**) current density distributions on the axial section in different schemes at different voltages; (**e**) temperature distribution on the axial section in inward scheme at 6 KV; (**f**) temperature distribution in the inside layer in outward scheme and outside layer in inward scheme at different voltages.

In order to quantitatively analyze the influence of the ECPT in different schemes, the FE (Finite Element) simulation was performed using the commercial FE software ANSYS platform. As shown in Fig. 5a,b, the copper coils were fixed inside and outside the tube samples, respectively, which were similar to the ECPT experiments in outward-discharging scheme and inward-discharging scheme. The copper coils in the ECPT experiments were fabricated by coiling the copper wire with square section, and only small gaps existed between neighboring copper wires, so the copper coil was simplified as a cylindrical tube in the FE models, which could reduce the complexity of FE simulation. According to the exciting current waveforms in group A and B (see Fig. 1e), the corresponding approximate formulas of the exciting currents 80e^−15400*t*^sin(700*t*) KV and 120e^−15400*t*^sin(700*t*) KV, where *t* was discharging time, were obtained by the fitting method. The fixed supports were applied on the outer surface of tube specimen in outward-discharging scheme and inner surface of tube specimen in inward-discharging scheme, respectively. During ECPT in inward-discharging scheme at 6 KV, the distribution of eddy current density induced within the tube specimens at t = 90 μs is shown in Fig. 5c. The distribution of eddy current density on the axial section of different specimens at t = 90 μs is shown in Fig. 5d, wherein the curves A, C represent the eddy current densities in the outside layer of axial section in inward-discharging scheme and B, D represent the eddy current densities in the inside layer of axial section in outward-discharging scheme. The highest eddy current density occurred in the outside layer in inward-discharging scheme and the inside layer in outward-discharging scheme, respectively, since the two layers were more close to the copper coil in their corresponding ECPT schemes. It indicates that the eddy current density was smaller in the middle zone and greater at both the ends of tube specimens because the tube specimen was shorter than the copper coil in this study. The density distribution of curve C was a little higher than the curve D at 6 KV discharging voltage, which was due to higher magnetic flux density inside the copper coil than outside^23^. When the discharging voltage increased to 9 KV, both the eddy current densities in outward-discharging and inward-discharging schemes increased pronouncedly, and the eddy current in inward-discharging scheme (see curve A) was higher than that in outward-discharging scheme (see curve B).

The temperature distribution at t = 90 μs on the axial section of the tube specimen in inward-discharging scheme at 6 KV is illustrated in Fig. 5e. The maximum temperature appeared at the both ends of axial section as a result of the highest eddy current density over there. The temperature distribution in the inside layer of axial section in outward-discharging scheme and the outer layer in inward-discharging scheme was compared in Fig. 5f. The result indicates that the maximum temperature increased with the increase of eddy current density, so the maximum temperature in inward-discharging scheme was a little higher than in outward-discharging scheme due to the difference of eddy current density in the two schemes, and higher discharging voltage led to higher maximum temperature on the axial section of tube specimens.

**Figure 6:**
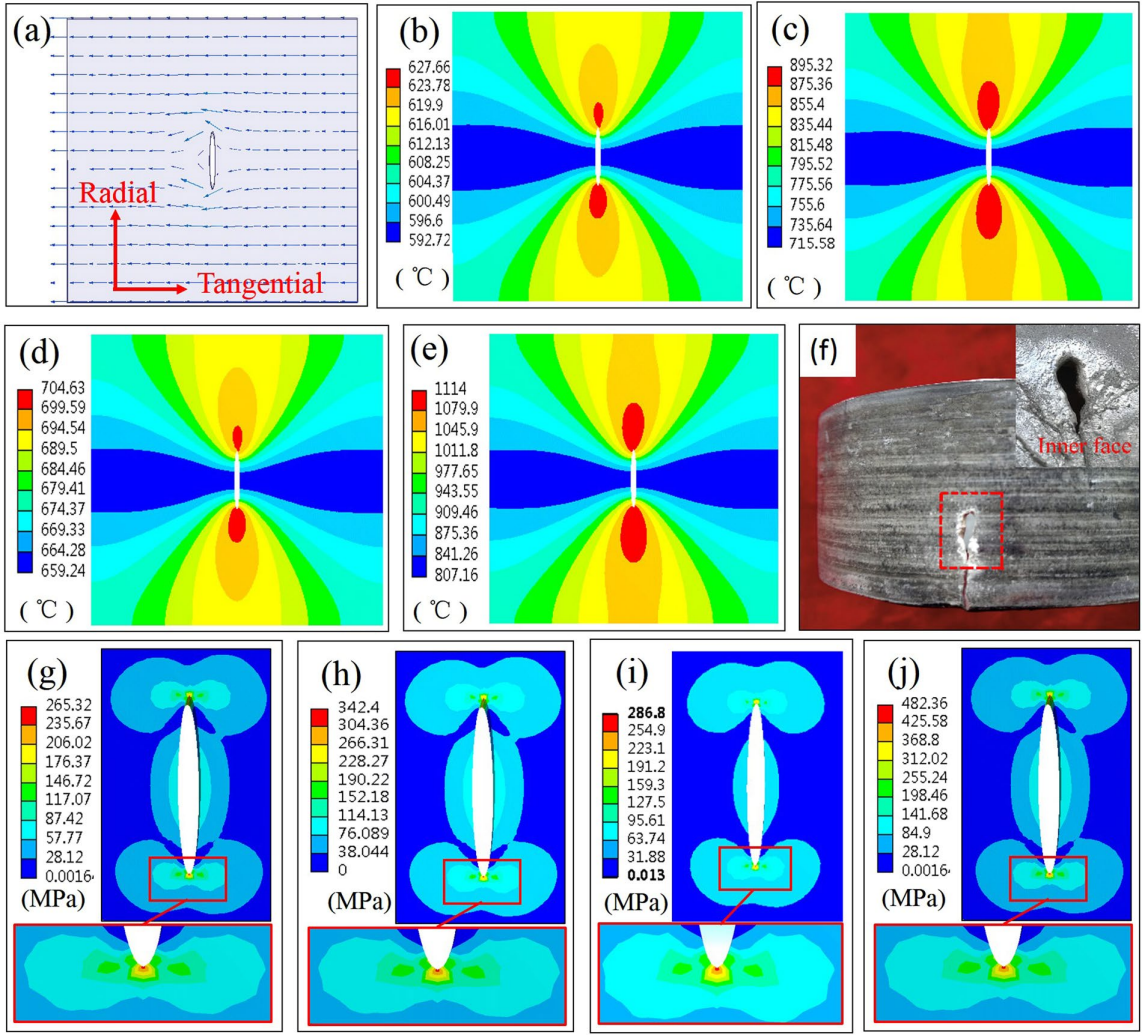
Simulation results of ECPT (t = 90 μs): (**a**) eddy current distribution; (**b**–**e**) temperature distribution around the microcrack in outward scheme at 6KV (b), 9KV(**c**) and in inward scheme at 6 KV (**d**), 9 KV (**e**); (**f**) tube specimen after ECPT at 11 KV; (**g**–**j**) equivalent stress distribution in outward scheme at 6 KV (**g**), 9 KV (**h**) and in inward scheme at 6 KV (**i**), 9 KV (**j**).

Since high density current pulse could heal the cracks in stainless steel^12,13^, 1045 steel^14,15^ and titanium alloy^16^ due to melting and compressive stress in the crack tips, high density eddy current pulse probably causes the melting and compressive stress in the crack tips. Therefore, the distribution of temperature and stress fields around the microcrack was analyzed through FE simulation. Generally, the microcracks in materials were assumed to be elliptic, which could describe a microcrack system more exactly^24^. In this study, the microcrack was simplified as an ellipse with a 0.1 mm major axis and 0.01 mm minor axis and built in the center of a rectangle cube (0.5 × 0.5 × 0.1 mm). The current density of 1.1 × 10^10^ A/mm^2^, 1.2 × 10^10^ A/mm^2^, 1.6 × 10^10^ A/mm^2^ and 1.8 × 10^10^ A/mm^2^, which were the maximum value of eddy current density among the present ECPT experiments in outward and inward scheme at 6 KV and 9 KV (see Fig. 5d), respectively, were set to flow in the direction perpendicular to the microcrack. According to the simulation results, the distribution of eddy current in Fig. 6a indicates that the eddy current detoured around the tip of microcrack. The concentration of eddy current around the microcrack tips led to the heat accumulation in the vicinity of microcrack tips, which was responsible for the temperature distribution. As shown in Fig. 6b and d, the maximum temperature in microcrack tips could reach 627.66 °C and 704.6 °C at 6KV in outward and inward-discharging scheme, respectively, which was much higher than the temperature in neighboring area. In Fig. 6c and e, the increase of discharging voltage raised the maximum temperature in the crack tip, which reached 895.32 °C in outward scheme and 1114 °C in inward scheme (at 9 KV), so metal softening and even melting may occur in the microcrack tips. For instance, when the 11 KV discharging voltage was applied in the ECPT experiment, some areas of tube specimen were melt and a hole appeared on the wall of tube specimen due to too much heat accumulation, as depicted in Fig. 6f. As can be seen from Fig. 6g, the equivalent stress at the crack tips could reach 265.3 MPa in outward scheme, and the tangential component of thermal stress σ_t_ (perpendicular to the length of microcrack in Fig. 6g) was 216.61 MPa. The tangential component of thermal stress σ_t_ could squeeze the two sides of microcrack in the microcrack tips, so the thermal stress played an important role in crack healing, especially in the crack tips. When the discharging voltage was raised to 9 KV, the maximum value of equivalent stress increased to 342.4 MPa in Fig. 6h, and the tangential component of thermal stress σ_t_ reached 264.87 MPa. In inward scheme shown in Fig. 6I,j, when the discharging voltage increased from 6KV to 9KV, the maximum value of equivalent stress increased from 286.8 MPa to 482.36 MPa and the tangential component of thermal stress increased from 234.17 MPa to 364.8 MPa. Therefore, higher density eddy current generated at higher discharging voltage would cause higher temperature and compressive stress in the microcrack tips, which contributed to better healing effect in the microcrack tips within tube specimens.

**Figure 7:**
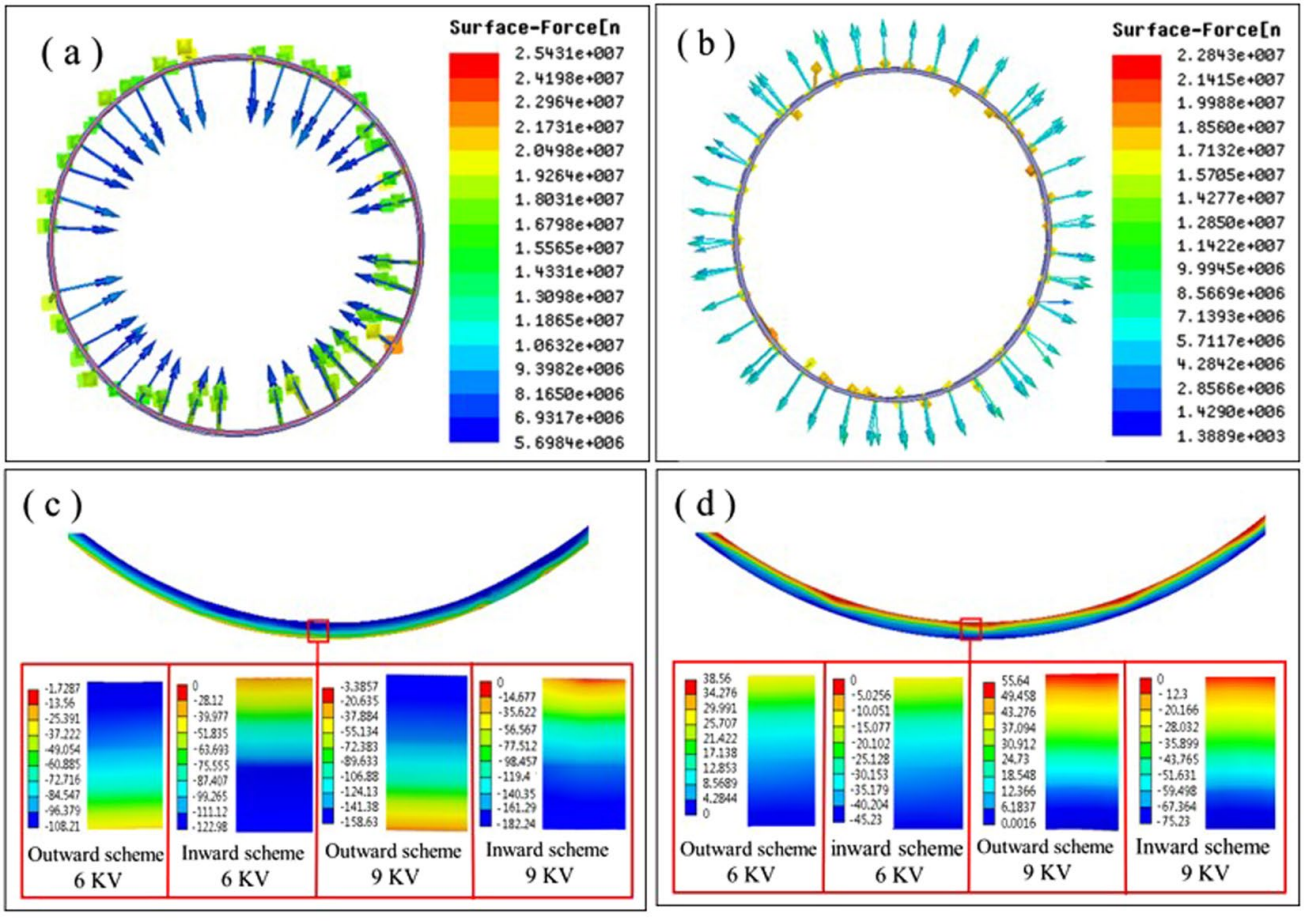
(**a**,**b**) Lorentz force distribution on the surface of tube wall in outward-discharging scheme (**a**) and inward-discharging scheme (**b**) at 6 KV. (**c**) radial stress distribution on the cross section in different discharging schemes with different voltages. (**d**) tangential stress distribution on the cross section in different schemes with different voltages.

From the SEM pictures of microcrack evolution, it should be noted that the microcrack closure was not so evident in tube specimen A4 after first 5 cycles of ECPT (see Fig. 2e,f), while more microcracks tended to be closed in tube specimen A5 (see Fig. 2i,j). As mentioned above, the tube specimen A4 and A5 were applied by ECPT under same discharging voltage 6 KV, which means that the energy consumptions of ECPT in the two schemes were very close. Actually, the simulation results of eddy current density distribution in Fig. 5d suggests that the eddy current densities of tube specimens in inward-discharging schemes with discharging voltage of 6 KV was a little higher than that in outward scheme. In addition, the relative positions of copper coil and tube specimen in outward and inward schemes were different. The distribution of Lorentz force on the surfaces of tube specimens in outward scheme and inward-discharging scheme at 6 KV are illustrated in Fig. 7a,b, respectively. According to the Faraday Law of electromagnetic induction^25^, the electromagnetic field could induce the Lorentz force, which was an inward radial force in the specimen A5 (in inward scheme) and an outward radial force in the specimen A4 (in outward scheme) during the ECPT. The inward radial force would squeeze the material towards the center of tube specimen, while the outward radial force would expand the material outwards. Furthermore, both the tube specimen A4 and A5 were tightly fixed during ECPT to prevent axial deformation, so the sidewall of tube specimen should be squeezed by Lorentz force, which would push the two sides of microcracks closer. Clearly, this squeezing force should be one of the driving forces for microcrack healing.

Since it was proportional to the eddy current density, the Lorentz force on the walls of the tube specimens increased with the increase of discharging voltage. Correspondingly, the stress caused by the Lorentz force would increase in the tube specimens. The distribution of radial stress and tangential stress on the cross section, which were caused by the Lorentz force, was illustrated in Fig. 7c,d, respectively. The maximum radial stress and tangential stress in outward scheme appeared in the inner layer of cross section, while the maximum values in inward scheme occurred in the outer layer of cross section. The maximum radial stress in inward scheme was a little higher than in outward scheme, and the radial stress was much higher than tangential stress at the same position. The radial stress squeezed both sides of tangential microcrack closer during the ECPT and more cycles of ECPT repeated this process, resulting in the improvement of micrcocrack healing degree. The increase of exciting current increased the radial stress, which could enhance the squeezing action on the two sides of microcracks, leading to better healing effect and mechanical performance. In inward scheme, the tangential stress also compressed the two sides of radial microcracks due to the inward Lorentz force, but the tangential stress may stretch the two sides of radial microcracks owing to the outward Lorentz force in the outward scheme. Therefore, the better healing effect and mechanical performance occurring in inward scheme than in outward scheme should be partly ascribed to different directions of the tangential stress, which was caused by the Lorentz force as well as slightly higher radial stress in inward scheme than in outward scheme.

**Figure 8:**
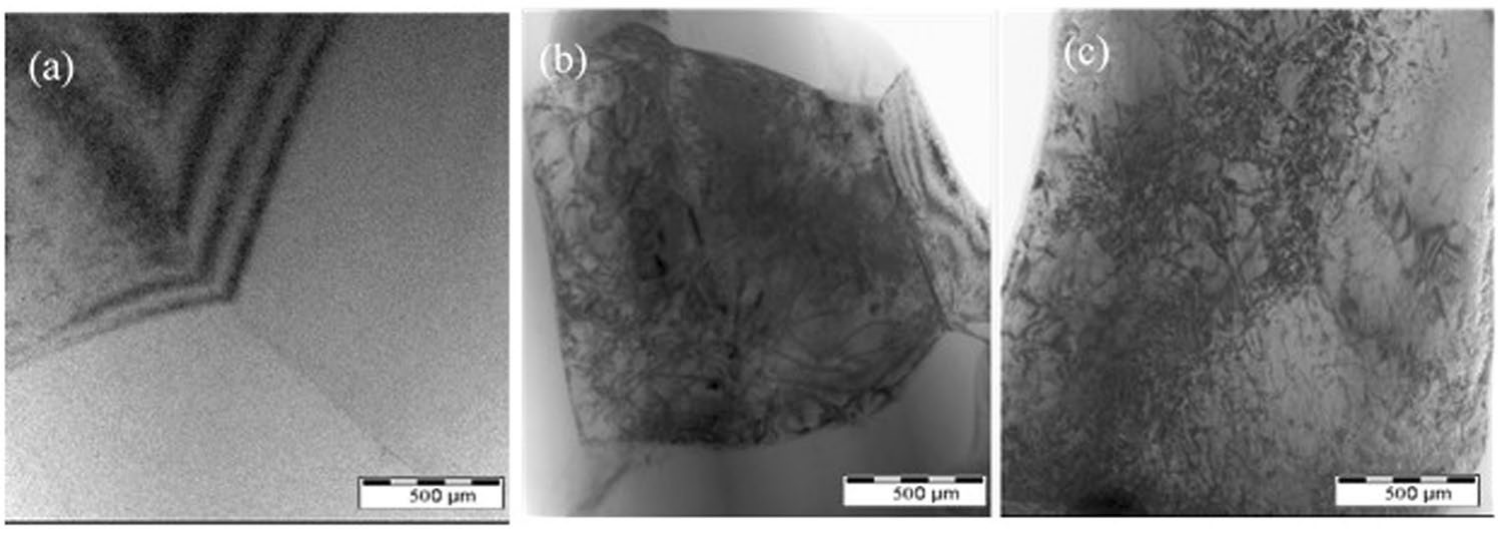
TEM images of A1, A4 and A5 after ECPT.

Although both of tube specimen A4 and A5 were tightly fixed by the stainless holder and stainless shaft, respectively, in the ECPT experiment, micro plastic deformation took place since the Lorentz force was exerted along the radial direction of tube specimens, which was similar to the compression and expansion of tubular workpieces in electromagnetic forming^26^. Therefore, the dislocation density in as-spun tube specimen was dramatically increased after ECPT, as shown in Fig. 8. It suggests that micro plastic deformation induced by ECPT might also contributed to the enhancement of the mechanical properties of magnesium alloy tubes.

**Figure 9:**
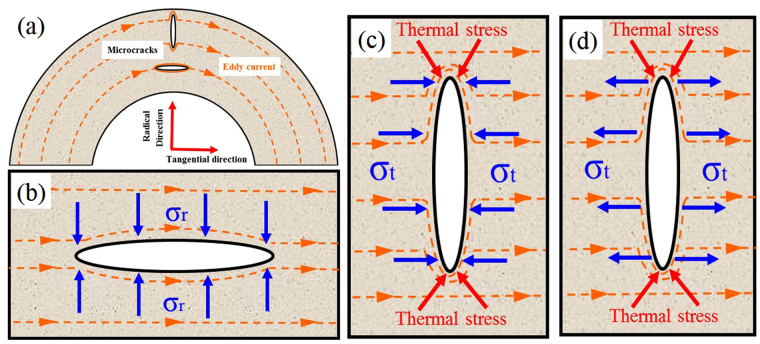
(**a**) The radial and tangential microcracks on the cross section. (**b**) The stress distribution around the tangential microcrack. (**c**,**d**) The stress distribution around the radial microcrack in inward scheme and outward scheme.

All in all, the mechanisms of microcrack healing during ECPT could be attributed to the thermal stress caused by the detouring of eddy current and the Lorentz force. Here, the radial microcrack and tangential microcrack will be discussed respectively. For the microcrack along the tangential direction shown in Fig. 9b, the radial stress caused by the Lorentz force σ_r_ squeezed the two sides of microcrack. However, the detouring of eddy current around the microcrack was not so pronounced, and the thermal accumulation in the vicinity of microcrack was relatively weak, so the thermal stress around the microcrack was too small to heal microcracks compared to the radial stress σ_r_. When the microcrack was distributed along the radial direction, the detouring of eddy current around the microcrack was quite evident and the thermal accumulation in the vicinity of microcrack was relatively remarkable. Besides, the tangential stress σ_t_ caused by the Lorentz force in outward and inward scheme was orientated along opposite directions: it compressed the two sides of microcrack in inward scheme and streched the two sides of microcrack in outward scheme (see in Fig. 9c,d). So in inward scheme, the microcrack along the radial direction could be successively healed under the combination of thermal stress and tangential compressive stress induced by the Lorentz force, as shown in Fig. 9c. But in outward scheme, it can be seen from the Fig. 9d that the tangential stress σ_t_ should prevent the closure of microcrack. Because the tangential stress σ_t_ was smaller than the tangential component of thermal stress, the microcrack could also be healed step by step. For instance, the FEA results show that when the tube specimen was subjected to the ECPT at 6 KV, the maximum tangential component of thermal stress reached 234.17 MPa, which was much higher than the tangential stress caused by the Loretz force (38.56 MPa), as shown in Fig. 7d. Although the distribution of microcracks in as-spun tube specimens was more complex (see Figs 2 and 3), both the thermal stress and Lorentz force could contribute to the healing of microcracks in tube specimens under enough cycles of ECPT, as indicated in the ECPT experiments.

According to the experimental and FEA results, the ECPT is an effective way to heal the microcracks automatically in the non-ferromagnetic alloys tubes, which only needs simple fixture tools and can work safely and effectively without directly contacting the tubular specimens. After ECPT, the mechanical performances of tubes were improved evidently without obvious microstructure change. Moreover, this method is not limited by the length of tubular workpieces. Hence, the ECPT process can be considered as a promising method for healing microcracks in non-ferrous alloy tubes.

## Conclusion

In this study, the ECPT method was firstly proposed for healing microcracks in non-ferromagnetic alloy tubes through the high density pulse power source supply. The experimental results indicate that the microcracks within as-spun magnesium alloy tube could be healed by ECPT, and the healing degree was promoted with the increasing circles of ECPT. Correspondingly, the strength and elongation of tube specimens were greatly increased. The increasing of the cycles of ECPT and discharging voltage contributed to the improvement of crack healing degree and mechanical properties of tube specimens, while the improvement of mechanical properties may be attenuated under too many ECPT cycles. Local melting appeared in tube specimen of Mg alloy tube under over high discharging voltage owing to too much heat accumulation in the vicinity of microcracks.

The crack healing during ECPT was ascribed to not only the thermal stress around the microcrack tips induced by the eddy current detouring the microcrack tips and the softening or melting of metals in the vicinity of microcrack tips, but also the squeezing action acted by the Lorentz force. Compared to the outward scheme, the inward scheme induced slightly higher density of eddy current pulse, leading to more thermal accumulation and thus greater thermal stress detouring the crack tips within tube specimens. Besides, both the compressive radial stress and tangential stress induced by the Lorentz force contributed to more sufficient crack healing in inward-discharging scheme, while the compressive radial stress and tensile tangential stress weakened the crack healing in outward-discharging scheme, albeit the tangential stress was much smaller than the tangential component of thermal stress around the crack tips. Therefore, the inward-discharging scheme could improve the mechanical properties of tube specimens more evidently compared to the outward-discharging scheme. The ECPT exhibits the great potential for healing microcracks in non-ferrous alloy tubes.
